# Post-traumatic stress disorder moderates the relationship between trauma exposure and chronic pain

**DOI:** 10.1080/20008198.2017.1375337

**Published:** 2017-09-19

**Authors:** J. Siqveland, T. Ruud, E. Hauff

**Affiliations:** ^a^ Division of Mental Health Services, Akershus University Hospital, Lørenskog, Norway; ^b^ Institute of Clinical Medicine, University of Oslo, Oslo, Norway; ^c^ Regional Center of Violence, Traumatic Stress and Suicide Prevention, Oslo, Norway; ^d^ Oslo University Hospital, Oslo, Norway

**Keywords:** Trauma exposure, post-traumatic stress, chronic pain, treatment

## Abstract

**Background**: Trauma exposure and post-traumatic stress disorder (PTSD) are risk factors for chronic pain.

**Objective:** This study investigated how exposure to intentional and non-intentional traumatic events and PTSD are related to pain severity and outcome of treatment in chronic pain patients.

**Methods**: We assessed exposure to potentially traumatizing events, psychiatric diagnosis with structured clinical interview, and pain severity in 63 patients at a secondary multidisciplinary pain clinic at the beginning of treatment, and assessed level of pain at follow up. Exposure to potentially traumatizing events and PTSD were regressed on pain severity at the initial session and at follow up in a set of multiple regression analysis.

**Results**: The participants reported exposure to an average of four potentially traumatizing events, and 32% had PTSD. Exposure to intentional traumatic events and PTSD were significantly associated with more severe pain, and PTSD significantly moderated the relationship between trauma exposure and pain (all p < .05). The treatment programme reduced pain moderately, an effect that was unrelated to trauma exposure and PTSD.

**Conclusions**: Trauma exposure is related to chronic pain in the same pattern as to mental disorders, with intentional trauma being most strongly related to pain severity. PTSD moderated the relationship between trauma exposure and pain. While pain patients with PTSD initially report more pain, they responded equally to specialist pain treatment as persons without PTSD.

## Abstract (es)

**Planteamiento**: La exposición al trauma y el trastorno de estrés postraumático (TEPT) son factores de riesgo para el dolor crónico. Este estudio investigó cómo el TEPT y la exposición a acontecimientos traumáticos intencionales y no intencionales se relacionan con la gravedad del dolor y el resultado del tratamiento en pacientes con dolor crónico.

**Métodos**: Al inicio del tratamiento, evaluamos la exposición a acontecimientos potencialmente traumatizantes, el diagnóstico psiquiátrico con la entrevista clínica estructurada y la gravedad del dolor en 63 pacientes de una clínica para el dolor multidisciplinaria secundaria. En el seguimiento, se evaluó el nivel de dolor. La exposición a acontecimientos potencialmente traumatizantes y al TEPT había retrocedido en cuanto a la gravedad del dolor en la sesión inicial y en el seguimiento en un conjunto de análisis de regresión múltiple.

**Resultados**: Los participantes informaron de la exposición a un promedio de cuatro eventos potencialmente traumatizantes, y el 32% tenían TEPT. La exposición a eventos traumáticos intencionales y el TEPT se asociaron significativamente con dolor más intenso y el TEPT moderó significativamente la relación entre la exposición al trauma y el dolor (todos los valores p <0,05). El programa de tratamiento redujo el dolor moderadamente, efecto que no estaba relacionado con la exposición al trauma y el TEPT.

**Conclusiones**: La exposición al trauma está relacionada con el dolor crónico de modo similar que con los trastornos mentales, siendo el trauma intencional el que está más intensamente relacionado con la gravedad del dolor. El TEPT moderó la relación entre la exposición al trauma y el dolor. Mientras que los pacientes con dolor y con TEPT inicialmente refirieron más dolor, respondieron del mismo modo al tratamiento especializado del dolor como las personas sin TEPT.

## Abstract (zh)

**背景：**创伤暴露和创伤后应激障碍（PTSD）是长期疼痛的风险因素。这个研究考察暴露于人为和非人为的创伤事件和PTSD是如何和长期疼痛患者的疼痛严重程度、治疗结果相关联的。

**方法：**在二级综合疼痛诊所中招募63名病人，在治疗的开始阶段我们评估了可能的创伤事件和疼痛程度，并使用结构化访谈进行精神诊断；在追踪阶段再次评估了疼痛程度。可能创伤事件的暴露和PTSD在初始和追踪疼痛程度上进行了一组多元回归。

**结果：**被试平均暴露于四个可能创伤事件，其中32%患有PTSD。暴露人为创伤事件和PTSD和更严重的疼痛显著相关；PTSD显著调节创伤暴露和疼痛的关系（所有p < .05）。治疗过程稍微减少了疼痛，但和创伤暴露和PTSD没有关系。

**结论：**创伤暴露和长期疼痛的关系模式就像和精神疾病一样，人为创伤和疼痛程度的关联最强。PTSD调节了创伤暴露和疼痛的关系。患有PTSD的疼痛病人最初报告更多疼痛，但他们和其它没有PTSD的病人对专门疼痛治疗的反应没有差别。

## 1. Introduction

Exposure to a potentially traumatizing event (PTE) is related to numerous somatic and mental health problems (McFarlane, 2010), including chronic pain. Chronic pain, defined as pain lasting more than 6 months, is among the most common and most costly health problems in Europe (Leadley, Armstrong, Lee, Allen, & Kleijnen, 2012). The condition is difficult to treat (Gatchel, McGeary, McGeary, & Lippe, 2014) and often has a fluctuating but generally chronic course (Walitt et al., 2011). For some patient groups, in particular veterans, having *both* PTSD and chronic pain is highly prevalent (Kip et al., 2014). Further understanding of the relationship between trauma exposure and chronic pain is of high clinical relevance.

One of the hindrances for research on how trauma exposure is related to pain is the ambiguous meaning of the concept ‘trauma exposure’. The events defined as trauma exposure have changed over time and have come to include many perhaps heterogeneous events (Breslau, 2002; McNally, 2003; Weathers & Keane, 2007). Originally the study of psychological trauma exposure came from research on the mental consequences of accidents and war (Weisæth, 2002), while the interest in other types of trauma exposure emerged later. The relative importance of different types of trauma exposure for the development of PTSD was investigated in a recent study of normal populations and prison inmates in the US (Briere, Agee, & Dietrich, 2016). Briere and colleagues investigated the relationship between cumulative traumas of either interpersonal or non-interpersonal type and found that it was the exposure to multiple types of interpersonal trauma that was associated with the greatest risk for PTSD. In general, events where the perpetrator intentionally harms the victim, such as interpersonal violence, sexual abuse, torture, and terrorism, are more strongly related to health problems than non-intentional events such as accidents, natural disaster, and illness (Frazier et al., 2009).

While interest in the somatic symptoms related to different types of trauma has long roots, more precise evidence based models are of newer date. The biopsychosocial model of pain suggests that medical illness, individual life history, environmental factors, and social relationships are all important for understanding and treating chronic pain (Turk & Okifuji, 2011).

PTSD is, together with depression, one of the two mental disorders most strongly associated with chronic pain (McWilliams, Cox, & Enns, 2003). Numerous studies have reported a positive relationship between PTSD and chronic pain in general (Ouimette et al., 2004) and in many different chronic pain conditions including fibromyalgia (Cohen et al., 2002), back pain and headaches (Vedantham et al., 2001), chronic regional pain syndrome (Speck, Schlereth, & Birklein, 2016) and arthritis pain (Lauterbach, Vora, & Rakow, 2005).

While numerous studies have examined the relationship between PTSD and chronic pain, only a few studies have included patients with chronic pain as participants. A recent systematic review (Brennstuhl, Tarquinio, & Montel, 2015) included all studies on the relationship between chronic pain and PTSD in populations without any specific underlying somatic disorder and found 24 articles meeting the inclusion criteria. Of these articles, only one study (Otis et al., 2010) recruited participants from a pain management programme. These participants were all military veterans and the findings from this study might not be applicable to a civilian population. Therefore, while the numbers of studies investigating pain and PTSD are high, the number studying this relationship from the perspective of a chronic pain population seeking treatment is much lower.

In this study, we analyzed the relationship between trauma exposure, PTSD and self-reported pain in patients at the first and at a later visit to a multidisciplinary pain clinic. Based on previous research, we hypothesized that patients previously exposed to trauma, in particular exposure to intentional events, and having a PTSD diagnosis, would report more severe pain and a more negative outcome of pain treatment.

## 2. Methods

### 2.1. Participants and procedure

At their first visit to a specialized pain clinic, patients were invited to participate in this interview study on traumatic stress and chronic pain. Data collection took place on two weekdays over a period of one year, and all new patients to the clinic on these days were invited to participate. Approximately half of the potential participants who were invited to participate accepted the invitation. The most common reason given for not participating was lack of time. All participants were interviewed by the first author, a certified specialist in clinical psychology. All patients with an adequate understanding of the Norwegian language were included. Written informed consent was collected from all participants prior to the interview, and all participants received 200 NOK (25 €) in compensation. The study was conducted in accordance with the Helsinki declaration of 1975 and approved by the Regional Committee for Medical and Health Research Ethics Health Region South-East (ID: 2010/1646a).

### 2.2. Measurement

Medical diagnosis and background demographic information were collected from electronic patient records.

#### 2.2.1. Trauma exposure

The participant’s level of exposure to PTEs was assessed with the Life Events Checklist (LEC). The LEC has 17 items with four response categories (0 = ‘not relevant,’ 1 = ‘confronted with,’ 2 = ‘witnessed,’ and 3 = ‘it happened to me’). Other studies have reported that the LEC has adequate reliability and validity (Gray, 2004). For further analysis, the LEC items were divided into two groups: exposure to intentional traumatic events such as interpersonal violence, sexual abuse, and war; and experiences related to non-intentional events such as accidents, natural disaster, and illness. The trauma exposure scale is an additive scale with no assumptions about the internal consistency of the scale, and hence no measure of internal reliability was calculated.

#### 2.2.2. Psychiatric diagnosis

The Mini-International Neuropsychiatric Interview (MINI 5.0.0, Norwegian adaptation) is a structured diagnostic interview assessing Axis I ICD-10 psychiatric disorders (Sheehan et al., 1998). The Norwegian version of the MINI has been validated previously and was found to have adequate reliability (Mordal, Gundersen, Bramness, & Gundersen, 2010).

#### 2.2.3. Pain

Pain severity at the start of treatment was assessed with a composite scale based on five questions assessing the current, highest, and lowest levels of pain, perceived burdensomeness of pain, and generalization of pain. The current, highest, and lowest levels of pain within the last week and perceived burdensomeness of pain were assessed by self-report on a 10-point visual analogue scale (VAS). Generalization of pain was also measured by self-report, where the participants marked where they had pain on a pre-drawn body picture; head/neck, chest, arms, stomach, upper back, lower back and legs. The number of pain sites was coded (0–7 with one point given for each of the pain sites).

Participants with valid responses to three of these questions were included, and missing values were replaced with imputed values for 14% of the participants with a multiple imputation algorithm in SPSS for Windows version 21. The pain severity scale had adequate internal reliability, with a Cronbach’s alpha of 0.76.

### 2.3. Analysis

Means and standard deviations were calculated for all variables. To analyze relationships between exposure to PTEs, PTSD, and pain, *t* tests and correlational and multiple regression analyses were conducted. The effect size of treatment was calculated as Hedges’ *g*. When 95% confidence intervals (95% CIs) were calculated, we used a bootstrapping procedure with 1000 iterations. All analyses were performed using SPSS version 15.

## 3. Results

Sixty-three participants consented to participate in the study, and 42 of these participants were available for follow-up. The remaining participants not included in the follow-up analysis had terminated their contact with the pain clinic within the first few sessions. These patients either received continued pain treatment from their primary care physician or elsewhere, or were not available for follow-up inclusion for other reasons. At baseline, the participants were between 24 and 66 years old (*M *= 44.9, SD = 10.5) and nearly two-thirds (64.5%) were women (*N* = 40). Among the participants, 27% received disability pension, 27% received monetary support for rehabilitation, 6% worked part-time, 33% worked full-time, and 6% were retired. As for education, 67% had finished high school and 16% had more than 3 years of college education. The participants had a range of different pain conditions. The most common was generalized pain: 26 participants had generalized pain, 21 had neuropathic pain, 9 had pain related to head or cervical pain and 7 had back or low back pain.

We first investigated the prevalence of trauma exposure, divided by sex (Table 1). The participants reported being exposed to an average of four PTEs with significantly more women than men reporting exposure to sexual abuse, war, and the death of a close one (*p* < 0.05).

**Table 1.** Prevalence of trauma exposure by gender.

Type of event	% reported having experienced	Men (*N* = 16)	Women (*N* = 26)
1. Natural disaster	2.3	6.3	3.7
2. Fire/explosion	16.3	12.5	18.5
3. Traffic accident	30.2	37.5	25.9
4. Accident at home/work	25.6	25.0	25.9
5. Exposure to poison	7.0	12.5	3.7
6. Physical attack	39.5	37.5	40.7
7. Assault with weapon	23.3*	18.8	25.9
8. Sexual abuse	37.2*	18.8**	48.1
9. Other sexual experience	11.6	6.3	14.8
10. War zone or battle experience	10.2*	6.3	3.7
11. Been taken hostage/POW	4.7	6.3	3.7
12. Life threatening disease/injury	34.9	43.8	29.6
13. Human suffering	9.3	12.5	7.4
14. Violent death	7.0	0	0
15. Sudden death	32.6*	12.5**	44.4
16. Caused injury other	11.6	18.8	7.4
17. Other stressor	23.3	25.0	22.2

*Significantly univariately related to PTSD, p < .05.

**Significant gender difference, p < .05.

Exposure to 4 of the 16 PTEs (assault with a weapon, war zone experience, sexual abuse, and sudden death) were significantly related to PTSD (*p* < 0.05).

As for mental disorders, depression was most common – 42% of the participants had a current major depressive disorder. About a third (32.3%) of the participants had PTSD, and PTSD was significantly more common among women than among men (*χ^2^* (1, *N* = 63) = 8.4, *p *< 0.05). Of those with PTSD, 55% were also depressed compared to 36% among participants without PTSD, a difference that did not reach statistical significance (*χ^2^* (1, *N *= 63) = 2.63, *p* = 0.105). Other mental disorders were less frequent: 21% had any anxiety disorder (excluding PTSD) and 6% had an alcohol or substance abuse disorder.

Secondly, we analyzed the bivariate correlation between a composite score of trauma exposure, divided into intentional and non-intentional events, PTSD, and pain (see Table 2).

**Table 2.** Sample characteristics and correlations.

	Mean and SD/					
	prevalence	1	2	3	4	5
1. Pain severity	5.6 (1.3)	*				
2. Trauma exposure total	13.4 (8.6)	.21*	*			
3. Intentional trauma	5.8 (5.7)	.29*	.82***	*		
4. Non-intent trauma	7.6 (5.1)	.14	.82***	.36***	*	
5. PTSD	32.3%	.36**	.38***	.49***	.18	*
6. Depression	41.9%	.31*	.06	.03	.09	.20

Bivariate *N*s range between 60 and 63; *p < 0.10, **p < .05, ***p < .01.

Overall trauma exposure was significantly related to PTSD (*r *= 0.40, *p* < 0.01) and pain severity (*r* = 0.26, *p* < 0.05). With trauma exposure divided into intentional and non-intentional events, only exposure to the intentional events was significantly related to PTSD (*r* = 0.49, *p* < 0.001) and to pain severity (*r* = 0.29, *p* < 0.05).

Our third analysis compared pain severity in persons with and without PTSD. We found that persons with PTSD reported more severe pain (*M *= 6.69, SD = 1.54) than persons without PTSD (*M* = 5.71, SD = 1.03), *t* (29.22), −2.64, *p* < 0. 05), and that PTSD was related to pain severity with a medium-to-large effect size (Hedges’ *g *= 0.80).

Our fourth analysis was a series of linear regression analyses assessing the direct and interaction effects of trauma exposure and PTSD on pain severity (see Table 3). In the first analysis we included two predictors; trauma exposure and PTSD. PTSD came out as the only significant predictor (*β* = 0.29, *p* < 0.05), and the model explained 13% of the variance in pain. In the second regression analysis, we included three predictors by dividing exposure into two: intentional and non-intentional trauma exposure and PTSD. This did not improve the model fit, and PTSD was the only predictor in this model. In the third regression analysis, we tested the effect of PTSD on moderating trauma exposure and pain by adding an interaction term (PTSD*exposure) to the regression.

**Table 3.** Multiple regression predicting severity of pain.

	Predictor	*β*	Std error	Sig	Adj *R*^2^
Model 1	Exposure	.18	.02	.18	.13
	PTSD	.29	.35	.03	
Model 2	Non-intentional exposure	.10	.03	.41	
	Intentional exposure	.11	.03	.44	
	PTSD	.29	.37	.04	.11
Model 3	Exposure	−.02	.02	.87	.21
	PTSD	.23	.31	.04	
	Exposure*PTSD	.41	.04	.01	

**Figure 1.**
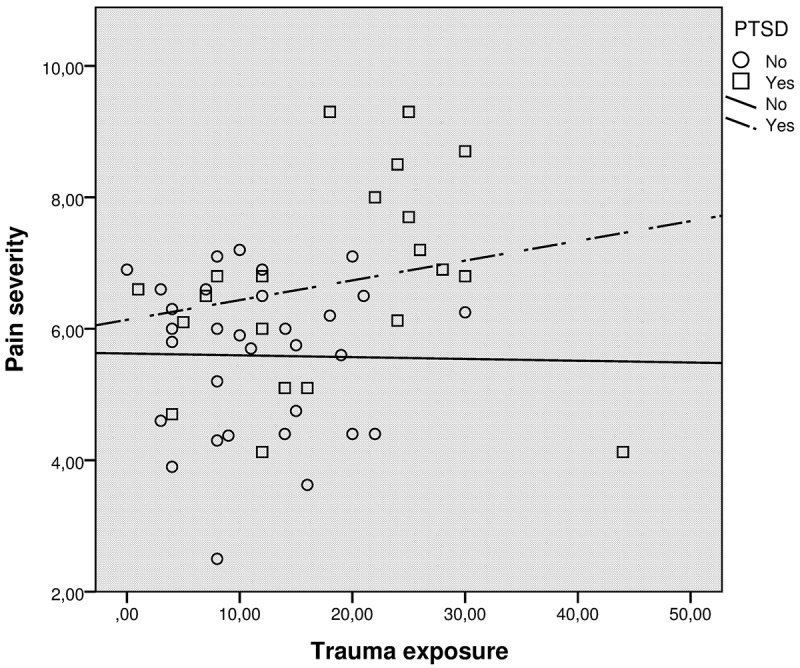
Relationship between trauma exposure and pain severity by PTSD. Footnote: r No-PTSD group = .03, p>.05; r PTSD group = .26, p>.05.

In this model, both PTSD (*β* = 0.23, *p* < 0.05) and the interaction term (*β* = 0.41, *p* < 0.05), came out as significant predictors explaining 21% of the variance in pain. The interaction effect between trauma exposure and PTSD for pain variance is presented in Figure 1 and shows that trauma exposure may be differentially related to pain depending on PTSD status. A Fisher r-to-z transformation to calculate the statistical significance of the difference in trauma exposure and pain correlations in the PTSD and the no PTSD groups (r = .26 and .03, respectively) (performed with an online calculator: http://vassarstats.net/rdiff.html) showed that these two correlations were not statistically significantly different (*z* = −0.82, *p* = 0.4122).

Our fifth analysis investigated the pain treatment outcome, and whether the treatment effect was related to PTSD. Pain levels, assessed on a 10-point VAS scale, were available from 42 participants (19 men, 14 with PTSD) and were collected an average of 17 months after the initial data collection. The mean level of pain in this group was 5.95 (SD = 1.90) at the beginning of treatment and 4.51 (SD = 2.22) at the end of treatment. The mean reduction in the raw pain score was (1.44, *t* (41) = 4.02, *p* < 0.001), indicating a moderate treatment effect (Hedges’ *g* = 0.69 (95% CI 0.25–1.13).

Lastly treatment outcome was analyzed in relation to PTSD status. Participants without PTSD (*n* = 28) reported average levels of pain at the start of treatment of 5.60 (SD 1.7) and at the end of treatment 4.25 (SD 2.07); i.e. an average raw pain score reduction of 1.35, Hedges’ *g* = 0.70 (95% CI 0.16–1.24). In participants with PTSD (*N* = 14), average pain levels at the start and end of treatment were 6.6 (SD = 2.1) and 5.04 (SD = 2.48), respectively; i.e. an average raw pain score reduction of 1.6, standardized effect size Hedges’ *g* = 0.66 (95% CI −0.10–1.42). Thus, there were no significant differences in treatment effects between persons with and without PTSD.

## 4. Discussion and conclusions

In these analyses we found that exposure to potentially traumatizing events that were intentional, were related to PTSD and more severe chronic pain. Further, PTSD moderated the relationship between trauma exposure and pain, and trauma exposure was related to more severe pain only in persons with PTSD. PTSD diagnosis was unrelated to the pain treatment outcome.

As hypothesized, we did find a difference in the risk of PTSD and more severe pain after exposure to intentional compared to non-intentional events; only intentional events were significantly related to PTSD and more severe pain. This difference in trauma exposure outcome was previously reported in a review of PTSD trajectories in 35 studies (Santiago et al., 2013). While exposure to both intentional and non-intentional events initially was related to an equal, and high, risk of symptoms of PTSD, they differed in their long-term trajectories of symptom maintenance. Persons exposed to non-intentional events experienced more rapid symptom recovery compared to persons exposed to intentional events. These differences are probably related to several factors. Exposure both to intentional and non-intentional events is related to worse mental health in a dose–response relationship (Park et al., 2014). Non-intentional stressors, such as traffic accidents and natural disasters, are typically shorter in duration compared to intentional events such as living with an abusive carer during childhood, or being a victim of interpersonal violence in an abusive relationship as an adult. Moreover, social factors commonly related to intentional events are related to a further risk of ill health due to a lack of protective and otherwise health promoting factors. A supportive family environment is important for post-trauma recovery (Maercker & Horn, 2013), and likely more available after traffic accidents compared with trauma related more to stigma, such as sexual abuse. While sex differences were not part of our original research hypothesis, we observed that women were exposed to intentional traumatic events more often than men, had a higher risk of PTSD, and reported higher levels of pain. This is in line with the finding that women reported more chronic pain (Moulin, Clark, Speechley, & Morley-Forster, 2002).

This weak-to-moderate positive relation between trauma exposure, PTSD and chronic pain is consistent with many previous studies of trauma exposure and pain patients where pain and other types of somatic complaints are more prevalent in trauma-exposed persons who develop PTSD. Vedantham et al. (2001) found in a study of Canadian bus drivers that trauma exposure was related to later somatic complaints only in persons with PTSD. Also, Andersen, Andersen, and Andersen (2014) found that persons with comorbid chronic pain and PTSD had significantly poorer health, poorer sleep quality, more cognitive problems and lower social functioning compared to pain patients without PTSD. While PTSD diagnosis has sometimes been heavily criticized for lacking in validity and clinical utility (McHugh & Treisman, 2007; Rosen, Spitzer, & McHugh, 2008), this study indicated that the PTSD diagnosis is useful in indexing persons who have the most severe health problems after trauma exposure. However our study is inconclusive on whether trauma exposure is more strongly related to pain in persons with PTSD even if we found a tendency towards this conclusion in our analysis.

This positive relationship between PTSD and pain was expected based on prediction of the shared vulnerability (Asmundson, Coons, Taylor, & Katz, 2002) and the mutual maintenance model (Sharp & Harvey, 2001). As previously mentioned, the mutual maintenance model suggests that the two conditions mutually maintain each other through an underlying positive feedback loop. According to this model, persons with PTSD experience exacerbating pain because they use avoidance as a coping strategy, have reduced activity levels, and attentional biases towards perceiving stimuli as implying danger. This model has received some empirical support among injured patients (Jenewein et al., 2009; Liedl et al., 2010) and in persons exposed to childhood abuse (Raphael, Spatz, & Widom, 2011).

While the participants reported a significant improvement in pain during treatment, the level of pain at the last visit to the clinic was still relatively high and further improvements in treatment options for this group are clearly needed.

One potential understanding of why trauma exposure is related to more severe pain was that pain is related to somatic injuries acquired during a trauma exposure such as a car accident. There is a number of reasons why we believe this explanation, while not formally investigated, is less likely. One reason is that the trauma exposure was seldom the cause of the pain. In the few instances where the pain was attributed to external causes it was more often related to minor injuries. For example, one person got chronic pain in one leg after jumping down from one metre height and spraining an ankle. The other reason is that the types of trauma that were related to more severe pain were interpersonal in nature, and more seldom led to lasting somatic injuries likely to cause pain, compared to events such as car accidents.

The issue of cumulative trauma exposure (Briere et al., 2016) may be important to understand the relationship between intentional and non-intentional events because these two types of events differ by two different qualities at the same time. There are two separate ways to understand how interpersonal events are uniformly related to more adverse health outcomes. One is that intentional events are inherently more dangerous for health; there is some qualitative difference between them that carries important differences for health. The other understanding of this differential relationship is that, while not part of the definition of the difference between them, an important difference is that of cumulative exposure. Many of the most common types of intentional events, such as violence within the family, are events the persons are exposed to numerous times and over long periods. The consequences of these types of traumas therefore are conferred through exposure compared to events such as accidents, which happen more rarely. The important difference between intentional and non-intentional events in this perspective is that the intentional events are related to higher exposure over longer time periods

The moderating role of PTSD, where trauma exposure was related to pain in persons with PTSD, is also in line with previous research by Raphael et al. (2011) which found that neither childhood victimization nor PTSD by themselves were related to increased risk of pain in adulthood. When the factors were combined, a significant relationship to pain was found. However, while the Raphael & Widom study investigated a population exposed to childhood abuse and neglect and not chronic pain patients, this finding is in line with our finding in these clinical pain patients. This finding is theoretically important because congruent with a PTSD diagnosis is a related propensity to experience chronic pain as suggested in previous theoretical models of the PTSD–pain relationship (Asmundson et al., 2002). Another interpretation of the importance of PTSD and its relationship to pain was suggested in a recent study of torture victims (Defrin, Lahav, & Solomon, 2016) where it is suggested that PTSD moderated the relationship between trauma exposure and pain by altering pain modulation.

In our follow up assessment we did not find that PTSD status at first assessment was related to worse outcome of treatment. This finding was, on the one hand, somewhat unexpected and partly contradictory to assumptions forming the mutual maintenance theory. According to the mutual maintenance theory, pain in persons with chronic pain should be less responsive to treatment because PTSD is supposed to be a driver for continued pain thorough a self-reinforcing cycle of arousal and avoidance (Sharp & Harvey, 2001). On the other hand this is also what Andersen et al. (2014) found in their study of chronic pain patients with PTSD.

Our last analysis investigated the outcome of pain treatment and whether this outcome was related to PTSD status. The treatment effect on pain was moderate to high and unrelated to PTSD status. Our finding that pain treatment was equally effective in reducing pain for persons with and without PTSD questions the clinical wisdom that PTSD limits the effectiveness of pain treatment (Outcalt et al., 2015). For a change in VAS score to be clinically (and not only statistically) significant, the change has to be around 1 point, with a 2-point difference indicating a more substantial change (Dworkin et al., 2008; Kelly, 2001); therefore, most participants on average had a clinically significant reduction in pain during this treatment. Unfortunately, we had no measure of PTSD symptomatology at the end of treatment, but any effect of pain treatment on PTSD symptoms is clearly of great clinical and theoretical interest for future research. However, with these limitations in mind, our study indicates that persons with PTSD might benefit from pain treatment to the same extent as non-PTSD patients. PTSD and pain, while both being common after trauma exposure, may be more separate phenomena, as recently suggested in a study of sexual assault survivors (Ulirsch et al., 2014).

We did not assess how many of our participants had been treated for their PTSD, but persons with PTSD may be reluctant to seek treatment because of the stigma related to a psychiatric diagnosis. The high prevalence of PTSD reported in many previous studies indicates that PTSD screening in chronic pain patients may be warranted for further referral. Screening in specialized pain clinics may facilitate PTSD treatment by motivating patients to seek PTSD treatment and make the necessary referrals. Some patients with co-morbid pain and PTSD may also benefit from treatment traditionally used for PTSD for the treatment of their pain symptoms. Recently Andersen, Lahav, Ellegaard, & Manniche (2017) tested the usefulness of adding a somatic experiencing (SE) component to treatment-as-usual (TAU) for a group of low back pain patients at a pain clinic. Patients receiving the SE treatment reported statistically lower levels of movement related fear compared to the patients in the TAU condition after treatment. While the study found a statistically significant difference between the groups, the difference was small and of uncertain clinical significance.

Some limitations are mentioned in the above discussion, but some merit more elaboration. We assessed the outcome of pain treatment but treatment effect compared to a control group not receiving treatment was not performed.

The limited sample size increased the risk of capitalizing on random fluctuations and the risk of both false positive and false negative findings (Button et al., 2013) as well as leaving some combinations of PTSD diagnosis and demographic background variables rare, such as men with PTSD. The study was introduced as a study on traumatic stress, which may have led to more persons with personal experiences of trauma exposure volunteering to participate. This may have overestimated the prevalence of PTSD in persons seeking treatment for chronic pain.

This investigation was based on the DSM IV criteria for PTSD. What possible difference it would mean to use the DSM V, were the criteria for PTSD substantially revised (Weathers, Marx, Friedman, & Schnurr, 2014), for this sample will have to remain speculative and based on what is generally known about prevalence differences between DSM IV and V. The main finding in that research is that the differences are minor and when they exist, the differences tend to be more persons meeting the diagnostic criteria according to the DSM V.

## 5. Conclusion

The present study suggests that both PTSD and some types of trauma exposure are related to severity of chronic pain. Clinicians treating pain patients should be observant of trauma history and PTSD status in their patients.

## Highlights of the article

- Exposure to potentially traumatizing events are common in persons seeking treatment for chronic pain
- Trauma exposure is related to more severe pain only in persons with PTSD
- PTSD may be particularly closely related to more generalized pain compared to more localized pain
- Persons with PTSD may respond equally well to specialist chronic pain treatment as persons without PTSD
